# Delayed breast feeding initiation increases the odds of colostrum avoidance among mothers in Northwest Ethiopia: a community-based cross-sectional study

**DOI:** 10.1186/s13690-021-00571-x

**Published:** 2021-04-07

**Authors:** Maezu G/slassie, Zelalem Nigussie Azene, Abuhay Mulunesh, Tesfa Sewunet Alamneh

**Affiliations:** 1 Department of Midwifery, College of Medicine and Health Sciences, Adigrat University, Adigrat, Ethiopia; 2 Department of Women’s and Family Health, School of Midwifery, College of Medicine and Health Sciences, University of Gondar, Gondar, Ethiopia; 3 Department of Epidemiology and Biostatistics, Institute of Public Health, College of Medicine and Health Sciences, University of Gondar, Gondar, Ethiopia

**Keywords:** Colostrum avoidance, Discard, Associated factors, Woman who gave birth in the last six months, Ethiopia

## Abstract

**Background:**

Colostrum is a yellowish and sticky breast milk produced in late pregnancy. Annually, 60% of 10.9 million under-five deaths globally are due to malnutrition. Of these, over two-thirds of the deaths are accounted by sub-optimal feeding practices in the first year of life, including colostrum discarding. However, evidence on the magnitude of colostrum avoidance and its associated factors at the community level is very limited in Ethiopia, particularly in the study area.

Thus, this study aimed to assess the magnitude of colostrum avoidance and associated factors among mothers who gave birth in the last six months in Gozamen district, northwest Ethiopia, 2019.

**Methods:**

A community-based cross-sectional study was conducted among 741 (741) mothers who gave birth in the last six months in Gozamen district from August 1 to September 12, 2019. A stratified cluster sampling technique was used to select the study participants. Data were collected by face-to-face interviewer-administered, pretested, and semi-structured questionnaire. Binary logistic regressions (bi-variable and multivariable) were fitted to identify statistically significant variables. Adjusted Odds Ratio (AOR) with 95% Confidence Interval (CI) was used to declare statistically significant variables on the basis of *p*-value < 0.05 in the multivariable binary logistic regression.

**Results:**

This study indicated that the magnitude of colostrum avoidance was 22.1% (95% CI, 19.0, 25.2%).

Mothers who did not get counseling on timely initiation of breast feeding (AOR = 3.91[95% CI, 1.98, 7.72]), not participate in pregnant woman forum (AOR = 2.59[95% CI, 1.30, 5.14]), initiate breast-feeding lately (more than 1 h) (AOR 2.27[95% CI, 1.18, 4.34]), and those having unfavorable attitude towards colostrum feeding (AOR = 7.35[95% CI, 3.89, 13.91]) were factors associated with the increased likelihood of colostrum avoidance. However, institutional delivery (AOR; 0.06[95% CI, 0.02, 0.19]) and prelacteal feeding (AOR; 0.10[95% CI, 0.05, 0.21]) were predictors associated with reduced likelihood of colostrum avoidance.

**Conclusion:**

Colostrum avoidance is a common practice in the study area. Therefore, in order to reduce this practice, strengthening infant feeding counseling, promoting institutional delivery, timely initiation of breastfeeding, health education, and community advocating are recommended interventions. In addition, creating awareness on the benefits of colostrum feeding is very instrumental to tackle the practice of colostrum avoidance.

## Background

Colostrum is a yellowish and sticky breast milk produced in late pregnancy and in the first few days after delivery [1]. World Health Organization (WHO) recommends colostrum as the first perfect food for newborns and feeding should be started immediately within the first 1 after delivery. It is considered as the first immunization for newborns and plays a great role in the immunological defense of newborn infants [2–4]. Colostrum feeding to a newborn is important to decrease the risk of undernutrition among young children [5]. Despite colostrum feeding provides newborns with immunity to infection, mothers in many developing countries still discard colostrum because of traditional beliefs such as viewing it as unclean and dirty milk having no nutritional value, to dilute to be useful, or seeing it as bad lucks for the family [6, 7]. Breast Feeding (BF) is the way of providing ideal food for infants to enhance their growth and development [8]. WHO and United Nations Children’s Emergency Fund (UNICEF) recommends breastfeeding immediately within 1 h after delivery, colostrum feeding, Exclusive Breast Feeding (EBF) for children up to six months of age, and sustained breastfeeding up to 24 months or above [2].

Colostrum avoidance (CA) is the discarding of colostrum during the first five days after birth [9]. Different studies indicated that sub-optimal breastfeeding like CA practice increases the risk of morbidities like diarrhea, pneumonia, Acute Respiratory Infections (ARI), and mortality among infants [10–12]. In addition, it increases the chance of Prelacteal Feeding (PLF) [13–15], barrier for Timely Initiation of Breast Feeding (TIBF), and Exclusive Breast Feeding (EBF) [6, 16, 17].

Globally, 60% of 10.9 million under-five children died every year directly or indirectly due to malnutrition and over two-thirds of the deaths are accounted for by inappropriate feeding practices that occurred in one year of life [2]. Sub-optimal breastfeeding in developing countries is responsible for 45% of neonatal deaths, 30% of diarrheal deaths, 18% of acute respiratory deaths in children [18, 19].

In Sub-Saharan Africa (SSA) and Southern Asia, under-five deaths are high which is 16 times more than in developed countries [20]. Furthermore, child mortality due to diarrhea is high in SSA countries including Ethiopia. Provision of colostrum for newborns, timely initiation of breast feeding, and exclusive breast feeding are among the preventive strategies of diarrheal disease in children [21]. Optimal breastfeeding practices including colostrum feeding in early life prevents about 13% of under-five deaths [22].

In Ethiopia, colostrum deficiency in early life was one risk factor for under-five stunting [23]. The practice of colostrum feeding is hindered by different factors including maternal health service utilization and neonatal factors [9, 24, 25]. Breast feeding is practiced in all parts of Ethiopia as a universal practice, but according to the mini Ethiopian Demographic Health Survey (EDHS) 2019, the prevalence of EBF at 6 months was 59%. There is still a gap in optimal breastfeeding practice [26] and in 2010, the national prevalence of colostrum avoidance was 39.8% [27]. Ethiopia has adopted the national Infant and Young Child Feeding (IYCF) guideline that discourages colostrum avoidance practice to attain optimal breastfeeding [28]. However, suboptimal feeding practice is documented after the implementation of the IYCF guideline. Colostrum avoidance is commonly practiced in different parts of Ethiopia. But, the reasons for it are not well studied in the country particularly in Gozamen district.

Furthermore, evidence on the magnitude of colostrum avoidance at the community level is very limited in Ethiopia. Therefore, the aim of this study was to assess the magnitude of colostrum avoidance and associated factors among mothers who gave birth in the last six months in Gozamen district, northwest Ethiopia.

## Methods

### Study design, setting, and period

A community-based cross-sectional study was conducted from August 1 to September 12, 2019, in Gozamen district. Gozamen district is one of the 18 districts in East Gojjam zone. Debre Markos is the zonal capital city of East Gojjam zone. It is located 300 kms away from Addis Ababa, the capital city of Ethiopia and, about 260 kms from Bahir Dar, the capital of Amhara regional state. The district is divided into 30 kebeles for administrative purpose (the smallest administrative unit next to the district in Ethiopia). Of these, 5 are urban and 25 are rural kebeles. According to the district administrative report, the population size of the district is estimated to be 164,816. Among these, about 82,573 are women, of those, 2536 are mothers who gave birth in the last six months. The district has now 6 health centers and 26 health posts providing health services to the population.

### Population

All mothers who gave birth in the last six-months in Gozamen district were the source population whereas those mothers who gave birth in the last six months in the selected kebeles/clusters of the district were the study population.

### Eligibility criteria

#### Inclusion criteria

All mothers who lived in Gozamen district for at least 6 months and gave birth in the last six months were included in the study.

#### Exclusion criteria

Those mothers who were seriously ill and unable to respond at the time of data collection were excluded from the study.

### Sample size determination and sampling procedure

The sample size was determined for the first and second objectives. Finally, the largest sample size was taken. Single population proportion was used to calculate the sample size for the first objective by considering the following assumptions:

Proportion = 12% [25], confidence level (CI), 95%, and margin of error = 4%.

Using the following single proportion formula:

$$ \frac{\mathbf{n}=\left(\mathbf{z}\boldsymbol{\upalpha } /\mathbf{2}\right)\mathbf{2}\ \mathbf{p}\left(\mathbf{1}-\mathbf{p}\right)}{\mathbf{d2}}\kern3em \frac{\mathbf{n}=\left(\mathbf{1}.\mathbf{96}\right)\mathbf{2}\Big(\mathbf{0.12}\left(\mathbf{1}-\mathbf{0.12}\right)}{\left(\mathbf{0.04}\right)\mathbf{2}}=\mathbf{253} $$

Where n = required sample size, Z = critical value for normal distribution at 95% confidence level (1.96), P = Proportion of colostrum avoidance, d = 0.04(4% margin of error). By considering a 10% non-response rate and a design effect of 2 the sample size was 560. For the second objective, the sample size was calculated using Epi info version 7.2 (double population proportion) using significant variables obtained from the previous study and the calculated sample size was greater than the first objective. Lastly, the larger sample size was taken (754). However, as stratified cluster sampling technique was employed to select the study participants the final sample size became 741. First, the population was stratified by residence as urban and rural Kebeles. The residential stratification gave 25 rural and 5 urban kebeles**.** Out of those, two urban and 7 rural kebeles were selected randomly by using a lottery method. Finally, all the selected kebeles were used as clusters, and all households in the selected Kebeles were included. Either of the mothers was interviewed in households having two mothers who gave birth in the last six months in the district. Households closed during the data collection period were revisited.

### Study variables and measurements

Colostrum avoidance is the response variable, whereas others like socio-demographic, maternal health service utilization, breastfeeding, obstetrical related characteristics, and maternal behavior-related variables are independent variables included in this study. Colostrum avoidance was considered as yes if the woman responded “**Yes**” when asked whether she discarded colostrum before giving breast to the infant within 5 days after delivery otherwise considered as no [9].

### Data collection tool and procedures

Data were collected using a pretested, semi-structured, and interviewer-administered questionnaire. The questionnaire was developed after reviewing relevant literatures. It was prepared originally in English and translated into the local language (Amharic) for the purpose of data collection and then it was translated back to English to maintain the consistency of the tool. The questionnaire has socio-demographic variables, health service utilization variables, obstetrics related variables, and maternal behavior related questions, breast feeding related variables and, wealth index related variables. For data collection, six Health Extension Workers (HEW) were involved under the supervision of the investigators and supervisors.

### Data quality control

A one-day training was given for data collectors and supervisors. The data were daily checked for completeness and accuracy by the principal investigator and supervisors.

A pretest was conducted on 5% of the sample size in the non-selected kebele of the district (Myanigetam) to ensure the validity, reliability, and clarity of the data collection instrument**.** Based on the findings from the pretest, modification on the questionnaire was done, and arrangement of questions was revised.

### Data analysis

Data were coded, cleaned, and entered into Epi data version 4.4.1 software. The data were exported to Statistical Package for Social Science (SPSS) version 20 for data analysis. Descriptive statistics (like median, interquartile range, frequencies, and percentages) were used to describe the study population in relation to dependent and independent variables. Results were presented in text, tables, and figures. Principal Component Analysis (PCA) was performed for urban and rural Kebeles separately to classify the household asset into low, middle, and high wealth indexes.

Binary logistic regression (bivariable and multivariable logistic regression) was used to identify statistically significant independent variables and independent variables having a *p*-value less than 0.2 in the bi-variable analysis were entered into multi-variable binary logistic regression. A *p*-value< 0.05 in the multivariable analysis was used to declare statistically significant variables. Hosmer-Lemeshow goodness-of-fit was used to test model fitness and declared good fitted at a p-value of > 0.05. An AOR with 95% CI was reported to show the strength of associations among the independent variables and colostrum avoidance.

## Results

### Sociodemographic characteristics of the respondents

A total of 741mothers who gave birth in the last six months were interviewed with a 100% response rate. The median age of the respondents was 30 years with (IQR ± 10 years) and the median age of their child was 3 months with (IQR ±3 months. Majority, (93.1%) of the respondents were married. Five hundred sixty-six (82%) of husbands’ occupation was farmer. Regarding educational status, 289 (41.9%) of husbands had no formal education. Three hundred seventy-eight (51%) of mothers had no formal education. Nearly two-thirds (65.7%) of the respondents’ occupation was a farmer. Almost all respondents (99.5%) and (99.6%) belongs to orthodox by religion and Amhara by ethnicit**y,** respectively (Table 1).

**Table 1:** Socio-demographic characteristics of mothers who gave birth in the last six months in Gozamen district, East Gojjam zone, North West Ethiopia, 2020 (*n* = 741)

Variables	Category	Frequency(***n***)	Percent (%)
Residence	Urban	98	13.2
	Rural	643	86.8
Sex of child	Male	343	46.3
	Female	398	53.7
Age of respondent	15–25 Years	162	21.9
	26–35 Years	375	50.6
	36–45 Years	204	27.5
Age of child	0–1 month	165	22.3
	2–3 month	290	39.1
	4–6 month	286	38.6
Family size	≤3	182	24.6
	≥4	559	75.4
Number of children	≤3	565	76.2
	≥4	176	23.8
Marital status of the woman	Single	26	3.5
	Married	690	93.1
	Widowed	9	1.2
	Separated	3	0.4
	Divorced	13	1.8
Educational status of the husband (*n* = 690)	Unable to write and read	289	41.9
	Able to write and read	206	29.8
	Primary school	129	18.7
	Secondary school	51	7.4
	College and above	15	2.2
Occupational status of the husband (*n* = 690)	Farmer	566	82
	Merchant	43	6.2
	Governmental and private employee	63	9.1
	Daily laborer	18	2.7
Occupational status of the woman	Housewife	175	23.6
	Merchant	22	3
	Private and governmental employee	31	4.2
	Farmer	487	65.7
	Daily labour	19	2.6
	Student	7	0.9
Educational status of the woman	Unable to read and write	378	51.0
	Able to write and read	176	23.8
	Primary school (1–8)	127	17.1
	Secondary school (9–12)	44	5.9
	College and above	16	2.2
Access to radio	Yes	417	56.3
	No	324	43.7
Access to television	Yes	60	8.1
	No	681	91.9
Household head	Yes	57	7.7
	No	684	92.3
Wealth index of family	Low	256	34.5
	Medium	238	32.2
	High	247	33.3

### Obstetrics and maternal behavioral related characteristics of the respondents

**Fig. 1:**
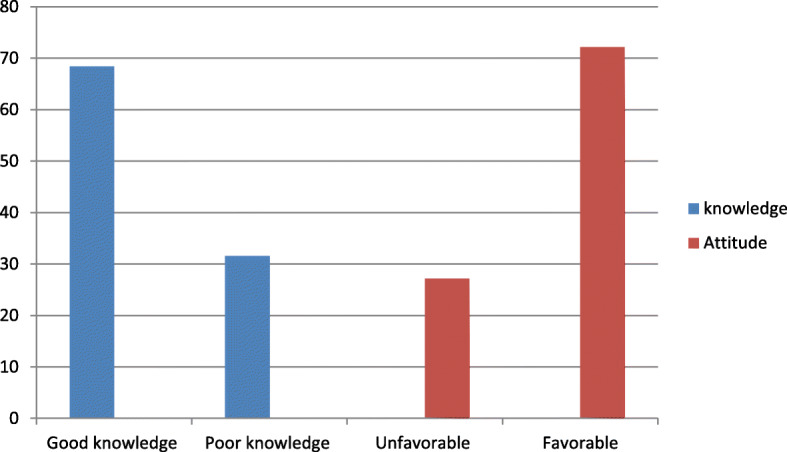
Knowledge and attitude of mothers who gave birth in the last six months about colostrum feeding in Gozamen district, East Gojjam zone, North West Ethiopia, 2020(*n* = 741)

Regarding parity, three-fourths (75.4%) of the study participants were multiparous, and more than half (58%) had optimal birth spacing (greater or equal to 24 months). About 508 (68.4%) of mothers had good knowledge about colostrum feeding and more than two-thirds of respondents (72.2%) had favorable attitude towards colostrum feeding (Fig. 1).

### Health care service utilization of the study participants

Concerning health care service utilization of the respondents, about 660 (89.1%) of the respondents had ANC follow-up. Out of these, 444 (67.3%) had less than four visits. Three hundred thirteen (47.4%) were counseled about breastfeeding. Of those, 121(38.7%) were counseled about exclusive breastfeeding. Majority of the study participants (88.9%) had institutional delivery. Out of the total respondents, 419 (56.6%) of mothers had participated in pregnant woman forum (Table 2).

**Table 2:** Health care service utilization of mothers who gave birth in the last six months in Gozamen district, East Gojjam zone, North West Ethiopia, 2020 (*n* = 741)

Variables	Category	Frequency(***n***)	Percent (%)
ANC visit (*n* = 741)	Yes	660	89.1
	No	81	10.9
Number of ANC visit (*n* = 660)	< 4	444	67.3
	≥ 4	216	32.7
BF counseling at ANC (*n* = 660)	Yes	313	47.4
	No	347	52.6
Counseling services	Benefits of breastfeeding	52	16.6
	Position during BF	22	7.0
	EBF	121	38.7
	Management of BF problems	13	4.1
	Expression of breast milk	18	5.8
	Colostrum benefits and should not discard	61	19.5
	Others*	26	8.3
Place of delivery (*n* = 741)	Health facility	666	89.9
	Home	75	10.1
Mode of delivery (*n* = 741)	Caesarian delivery	67	9
	Spontaneous vaginal delivery	659	88.9
	Instrumental delivery	15	2.1
Person assisted the labour (*n* = 741)	Health professional	657	88.7
	Traditional birth attendant	15	2.0
	Family (mother, husband)	56	7.6
	Others**	13	1.7
PNC visit (*n* = 741)	Yes	567	76.5
	No	174	23.5
BF counseling at PNC (*n* = 567)	Yes	263	46.4
	No	304	53.6
Participation in pregnant woman forum	Yes	419	56.5
	No	322	43.5

Key: Others* = start Bf within one hr., avoid PLF, and feed their child 8–12 time per day

Others** = mother, sister in law, and neighbor

### Breast feeding-related characteristics

One hundred sixty-four (22.1%) of the respondents discarded colostrum within five days after delivery. One hundred twenty-even (17.1%) of mothers had practiced pre-lacteal feeding. The most common pre-lacteal feeding was butter (81.1%) (Table 3).

**Table 3:** Breastfeeding-related characteristics of mothers who gave birth in the last six months in Gozamen district, East Gojjam zone, North West Ethiopia, 2020 (*n* = 741)

Variables	Category	Frequency(***n***)	Percent (%)
Colostrum avoidance (n = 741	Yes	164	22.1
	No	577	77.9
Reasons for colostrum avoidance (*n* = 164)	Causes abdominal cramp and diarrhea	42	25.6
	Dirty	38	23.2
	Cultural practice	25	15.2
	Maternal medical illness	13	7.9
	My breast has no milk	14	8.5
	Not good for child growth	5	3
	Infant not feed	9	5.5
	Influence by others	12	7.3
	Others*	6	3.8
Prelacteal feeding (*n* = 741)	Yes	127	17.1
	No	614	82.9
Types of prelacteal feeding (*n* = 127)	Butter	103	81.1
	Milk	19	15
	Water	5	3.9
Reasons for prelacteal feeding (*n* = 127)	Breast milk causes thirsty	3	2.4
	Good for child growth	9	7.1
	Breastfeeding problem	17	13.4
	Maternal medical illness	18	14.2
	Cultural practices	22	17.3
	To calm baby	7	5.5
	To clean bowel and throat	44	34.6
	Others**	7	5.5
Time of initiation of breastfeeding (*n* = 741)	More than 1 h	290	39.1
	Within 1 h	451	60.9
BF counseling on timely initiation	No	293	39.5
	Yes	448	60.5

Key: Others* = causes heart disease and constipation

Others** = to prevent constipation, belief BF is not enough

### Factors associated with colostrum avoidance

Bi-variable and multi-variable binary logistic regression were done to identify factors associated with colostrum avoidance. In bi-variable analyses, age of women, husband’s occupation, access to TV, breastfeeding counseling during ANC, place of delivery, PNC visit, breastfeeding counseling on timely initiation, participation in pregnant woman forum, prelacteal feeding, time of initiation of breastfeeding, and attitude towards colostrum had association with colostrum avoidance. Only participation in pregnant woman forum, place of delivery, time of breast feeding initiation, pre-lacteal feeding, breastfeeding counseling on timely initiation, and attitude of mothers towards colostrum were significantly associated with colostrum avoidance in multi-variable binary logistic regression analysis.

Mothers who did not get counseling on timely initiation of BF were 3.9 times more likely to avoid colostrum (AOR = 3.91[95% CI, 1.98, 7.72]) than those respondents who did get counseling. Women who failed to participate in pregnant woman forums were 2.59 times more likely to discard colostrum (AOR = 2.59 [95% CI, 1.30, 5.14]) as compared to their counterparts. The odds of colostrum avoidance were 2.27 times higher in individuals who initiated breastfeeding in more than one Hr. (AOR 2.27[95% CI, 1.18, 4.34]) as compared to their counterparts.

In addition, the odds of colostrum avoidance was 7.35 times higher in mothers who had an unfavorable attitude towards colostrum (AOR = 7.35[95% CI; 3.89, 13.91]) as compared to their counterparts. Respondents who gave birth at health facilities were 94% times less likely to discard colostrum (AOR; 0.06[95% CI, 0.02, 0.19]) as compared to mothers who gave birth at home. Those mothers who did not give pre lacteal feeding were 90% times less likely to discard colostrum (AOR; 0.10 [95% CI; 0.05, 0.21]) as compared to those respondents who gave prelacteal feeding (Table 4).

**Table 4:** Bivariable and multivariable logistic regression analysis showing factors associated with colostrum avoidance in Gozamen district, East Gojjam zone, North West Ethiopia, 2020(*n* = 741)

Variables	Colostrum avoidance Yes No	COR(95% CI)	AOR(95%CI)	***P***-value
**Husband occupation**				
Merchant	6 37	0.13 (0.36,0.46)	0.81 (0.07,8.66)	0.973
Farmer	120,446	0.22 (0.08,0.56)	0.62 (0.08,4.57)	0.641
Governmental and private employee	9 54	0.13 (0.04,0.43)	0.37 (0.04,3.44)	0.625
Daily laborer	10 8	1	1	
**Age of the respondent**				
15–25	29,133	0.65 (0.39,1.11)	0.88 (0.34,2.28)	0.401
26–35	85,290	0.88 (0.590,1.310)	1.29 (0.61,2.81)	0.741
36–45	51,153	1	1	
**Access to TV**				
No	159,522	2.74 (1.16,6.49)	1.58 (0.44,5.75)	0.618
Yes	6 54	1	1	
**BF counseling during ANC**				
No	76,271	2.30 (1.49,3.57)	1.28 (0.64,2.56)	0.793
Yes	34,279	1	1	
**Place of delivery**				
Health facility	99,567	0.02 (0.01,0.05)	0.06 (0.02,0.19)**	**< 0.00**
Home	66 9	1	**1**	
**PNC visit**				
No	57,117	2.07 (1.42,3.02)	0.87 (0.42,1.82)	0.280
Yes	108,459	1	1	
**BF counseling on timely initiation**				
No	132,161	10.31 (6.76,15.73)	3.91 (1.98,7.72)**	**< 0.001**
Yes	33,415	1	**1**	
**Participation in pregnant woman forum**				
No	136,186	9.83 (6.35,15.23)	2.59 (1.30,5.14)*	**0.013**
Yes	29,390	1	**1**	
**Prelacteal feeding**				
No	79,535	0.07 (0.05,0.11)	0.10 (0.05,0.21)**	**< 0.001**
Yes	86 41	1	**1**	
**Time of BF initiation**				
More than 1Hr	130,160	9.66 (6.38,14.63)	2.27 (1.18,4.34)*	**0.008**
Within 1Hr	35,416	1	**1**	
**Attitude towards colostrum**				
Unfavorable	90 79	7.55 (5.12,11.12)	7.35 (3.89,13.91)**	**< 0.001**
Favorable	75,497	1	**1**	

NB: * = *P* < 0.05, ** = *P* < 0.001, 1 = Reference category, Hosmer and Lemeshow goodness -of- fit = *P*- value = 0.844, *AOR* Adjusted odds ratio, *COR* Crude odds ratio, *CI* Confidence interval

## Discussion

Even though WHO, global and national IYCF guidelines recommended that all newborns should start breastfeeding immediately (within the first 1 h after delivery) and encouraged colostrum feeding [2], colostrum is still discarded in different parts of the globe, particularly in Ethiopia [6, 7]. Furthermore, evidence on the magnitude of colostrum avoidance at the community level is very limited in the country, especially in the study area. As such, the current study was conducted to determine the magnitude of colostrum avoidance and to identify the factors associated with it. This study showed that the magnitude of colostrum avoidance was found to be 22.1% (95% CI, 19, 25.2). This finding (22.1%) is higher than the studies conducted in rural areas of Mettu district 17.5% [29], Raya kobo district 13.5% [30], and Arbaminch Zuria11% [6]. This discrepancy might be due to socio-cultural and infant feeding styles that affect colostrum feeding since colostrum avoidance is traditional malpractice affected by the attitude and cultural beliefs of the community. In addition, this finding is higher than studies done in urban areas of Ethiopia such as; Axum town 6.3% [9], North Wollo zone 12% [25], Kombolicha town 11.4% [31], and Bahir Dar city 16.7% [32]. The possible explanation for the observed discrepancy could be due to differences among study settings. The earlier studies conducted in the urban parts of the country where there is better access to different information on infant feeding practices [33, 34].

The result of our study is in line with studies conducted in Motta 20.3% [35] and Mizan Aman teaching hospital 23.7% [36]. This similarity might be due to similar sociocultural practices between the two settings. However, the prevalence of colostrum avoidance in this study is lower than studies carried out in Kossaye, rural northern Ethiopia 79% [37], national prevalence of Ethiopia 38.9% [27], Afambo, Afar regional state 35% [5, 27], Jimma Arjo Woreda 27.5% [7], block RS Pura 76% [38], Bhavnagar city, Gujarat India 63.1% [39], Kamrup Assam, India 29.5% [40], rural health training center Santhiram medical college Nandyal 90.6% [12], Mansoura district in Egypt 57.8% [41], and South Sudan 38.8% [42]. The possible explanation may be due to the differences in the study population, socio-demographic characteristics, socio-culture, and year of study across studies.

Regarding factors associated with colostrum avoidance, mothers who did not get counseling on timely initiation of BF were 3.9 times more likely to discard colostrum as compared to their counterparts (AOR = 3.91[95% CI; 1.98, 7.72]). This is consistent with a study done in Kombolicha town [31]. The possible explanation for this finding might be due to the fact that mothers who did not get counseling on timely initiation of BF have no knowledge of when to initiate BF, thus, they are liable to initiate BF lately, when they initiate breastfeeding lately, they will have enough time to discard colostrum due to miss information on colostrum feeding.

This study also showed that mothers who did not give prelacteal feeding were 90% times less likely to discard colostrum as compared to those respondents who gave prelacteal feeding (AOR; 0.10 [95% CI; 0.05, 0.21]). This is supported by a study done in North Wollo zone [25]. The possible reason might be due to the fact that mothers who did not give prelacteal feeding have no other options than colostrum as food for their children [42].

Furthermore, late initiation of breast feeding was another factor found significantly associated with colostrum avoidance practice. Mothers who initiated breastfeeding in more than 1 h were 2.27 times more likely to discard colostrum as compared to their counterparts (AOR 2.27[95% CI; 1.18, 4.34]). This is consistent with studies conducted in North Wollo zone, Raya Kobo district, and systematic review done in Ethiopia [25, 30, 43], respectively. This might be explained as the time interval increases between delivery and breastfeeding initiation, there will be more time for infant feeding malpractices like colostrum avoidance [25].

The odds of colostrum avoidance was 2.59 times higher among mothers who did not participate in pregnant woman forum as compared to mothers who participated in pregnant woman forum (AOR = 2.59 [95% CI; 1.30, 5.14]). This may be due to the fact that participation in health education during pregnant woman forum will increase mothers’ awareness towards colostrum feeding. This finding is comparable with a study carried out in Kombolicha town [25].

This study also revealed that mothers who gave birth in health institutions were 94% times less likely to avoid colostrum as compared to mothers who gave birth at home (AOR; 0.06[95% CI; 0.02, 0.19]). This is in line with studies conducted in North Wollo zone, Bhavnagar city, Raya Kobo district, and Madhya Pradesh India [25, 30, 39, 44], respectively. This might be due to the fact that mothers who gave birth in health institutions might start breastfeeding immediately within 1 h after delivery and then have no time to discard colostrum. In addition, institutional delivery does not create a favorable environment for different sociocultural factors, like family influence. Besides, mothers who gave birth in health institutions receive advice from health professionals about the risks associated with colostrum avoidance which in turn helps them reduce the chance of colostrum avoidance [30, 34].

Once more, the odd of colostrum avoidance was 7.35 times higher in mothers who had unfavorable attitude towards colostrum as compared to mothers who had favorable attitude towards colostrum (AOR = 7.35[95% CI; 3.89, 13.91]). There is no study consistent with this finding as per our review. This might be due to the fact that colostrum avoidance is traditional malpractice more associated with belief and if people hold unfavorable attitude towards colostrum, the probability of discarding it will be high.

This study has some limitations that ought to be taken into account when interpreting the results. First, cross-sectional nature of this study may not show the temporal relationship between the dependent and independent variables. Second, ascertainment of colostrum avoidance was relied on the memory/information given by the study participants, which might be prone to recall bias.

## Conclusions

This study revealed that colostrum avoidance is a common practice in the study area. Place of delivery, breast feeding counseling on timely initiation, time of BF initiation, prelacteal feeding, participation in pregnant woman forum, and attitude of mothers towards colostrum were significant independent predictors associated with colostrum avoidance. In order to reduce this practice, strengthening infant feeding counseling, promoting institutional delivery, timely initiation of breastfeeding, health education, and community advocating are recommended interventions.

## Data Availability

The dataset analyzed during the current study available from the corresponding author on reasonable request.

## Abbreviations

ANC: Antenatal Care
AOR: Adjusted Odds Ratio
BF: Breast Feeding
CA: Colostrum Avoidance
CI: Confidence Interval
EBF: Exclusive Breast Feeding
IYCF: Infant and Young Child Feeding
PCA: Principal Component Analysis
PLF: Pre Lacteal Feeding
PNC: Postnatal Care
SPSS: Statistical Package for Social Science
SSA: Sub Saharan Africa
TIBF: Timely Initiation of Breast Feeding
UOG: University of Gondar
